# Mdivi‐1 induced acute changes in the angiogenic profile after ischemia‐reperfusion injury in female mice

**DOI:** 10.14814/phy2.13298

**Published:** 2017-06-02

**Authors:** Sudhakar Veeranki, Suresh C. Tyagi

**Affiliations:** ^1^ Department of Physiology and BiophysicsUniversity of Louisville School of MedicineLouisvilleKentucky40202

**Keywords:** Angiogenesis, arrays, heart, IR injury, Mdivi‐1, mitochondrial fission

## Abstract

The aim of this study is to determine the effects of mitochondrial division inhibitor 1 (Mdivi‐1), the mitochondrial fission inhibitor, on the angiogenic profiles after the ischemia reperfusion injury (IR injury) in female mice. Female mice were treated with Mdivi‐1 inhibitor, 2 days prior, on the day of IR injury and 2 days after IR injury, for a period of 5 days. Both control and treatment groups underwent 30 min of ischemia and 72 h of reperfusion. On the day 3, mice were sacrificed and the ischemic and nonischemic portions of heart tissue were collected. Relative levels of 53 angiogenesis‐related proteins were quantified simultaneously using Angiogenic arrays. Heart function was evaluated before and after 72 h of IR injury. Mdivi‐1 treatment ameliorated IR induced functional deterioration with positive angiogenic profile. The seminal changes include suppression of Matrix metalloproteinase (MMP3), tissue inhibitor of metalloproteases (TIMP1) and chemokine (C‐X‐C motif) ligand 10 (CXCL10) levels and prevention of connexin 43 (Cx43) loss and downregulation in the antioxidant enzyme levels. These changes are correlated with enhanced endothelial progenitor cell marker (cluster of differentiation (CD31), endothelial‐specific receptor tyrosine kinase (Tek), fMS‐like tyrosine kinase 4 (Flt4) and kinase insert domain protein receptor (Kdr)) presence. Our study is the first to report the role of mitochondrial dynamics in regulation of myocardial IR‐induced angiogenic responses. Inhibition of excessive mitochondrial fission after IR injury ameliorated heart dysfunction and conferred positive angiogenic response. In addition, there were improvements in the preservation of Cx43 levels and oxidative stress handling along with suppression of apoptosis activation. The findings will aid in shaping the rational drug development process for the prevention of ischemic heart disease, especially in females.

## Introduction

Myocardial infarction (MI) and cardiac ischemia/reperfusion injury (IRI), two different manifestations of ischemic heart disease, occupy the major share of the cardiovascular disease, which is the leading global cause of mortality and morbidity (Writing Group Members, 2016). During MI, blockade of the major coronary arteries that nourish myocardium results in ischemic myocardium leading to myocardial tissue death, thinning of the ventricle walls, fibrosis and heart failure. Ironically, reestablishment of perfusion after ischemia through angioplasty also leads to myocardial injury and significant myocardial death, referred to as ischemia reperfusion injury (IR injury). For MI and IR injury, there is still no effective therapy (Hausenloy and Yellon 2013; Hashmi and Al‐Salam 2015). In the recent past, there have been significant breakthroughs in deciphering the pathophysiology of IR injury. Failure of mitochondrial function has been identified as one of the key mechanisms in the causation of IR‐mediated myocardial damage/death (Hausenloy and Yellon 2013; Ong et al. 2015). Heightened oxidative stress and calcium overload gained prominence during IR‐mediated mitochondrial fragmentation and failure, which, if not stopped, leads to myocardial cell death (Ong et al. 2010; Kalogeris et al. 2012; Disatnik et al. 2013).

Balanced mitochondrial biogenesis and mitophagy are necessary for proper mitochondrial homeostasis, a key process in cell's survival and adaptation in response to stress such as IR injury. Fusion and fission processes regulate mitochondrial dynamics. Previous studies have unraveled the key mediators of fusion and fission. Mitofusin 2 (Mfn2) regulates mitochondrial fusion and dynamin‐related protein 1 (Drp1) regulates mitochondrial fission along with other participating molecules (Givvimani et al. 2014, 2015; Veeranki et al. 2016). Excessive/uncontrolled mitochondrial fission leads to fragmentation and mitophagy, which confers poor cell survival, especially during stress conditions. Elevated mitochondrial fission has been observed during IR injury and MI. Consequently, inhibition of mitochondrial fission regulator, Drp1, has been proposed as a viable therapeutic strategy (Ong et al. 2010). In this direction, a potent Drp1 inhibitor, mitochondrial division inhibitor 1 (Mdivi‐1), has been developed and was successfully employed in amelioration of IR injury (Ong et al. 2010) with the reductions in myocardial apoptosis and mitochondrial transition pore (MTP) opening as benefits of such treatment. Implications of MTP pore inhibition are enormous in the prevention of apoptosis, especially during IR injury. For example, MTP opening prevents cytochrome c release, leakage of antioxidant molecules (glutathione) and abnormal Ca^2+^ release, which are the prominent triggers of apoptosis (Hashmi and Al‐Salam 2015). Interestingly, abnormal Ca^2+^ cytosolic accumulation during IRI also activates calcineurin, which further activates Drp1 translocation to mitochondria and mitochondrial fission by dephosphorylation of the S637 inhibitory phosphorylation (Cereghetti et al. 2008). In spite of such progress, the full spectrum of changes associated with mitochondrial fission inhibition after IR injury remains largely unknown. Specifically, the modulations in the regulators of angiogenesis and microcirculation are unknown. Recently, it was demonstrated that proper mitochondrial function is crucial for stress‐induced transcriptional regulation and any attenuation in the mitochondrial function is bound to change gene expression profiles (Picard et al. 2015). This study has emphasized the significance of mitochondrial function/communication in transcription. Any perturbations in the commutation lead to abnormal cell responses and eventual maladaptation. Hence, preservation of mitochondria, by preventing excess mitophagy during the IR stress may lead to better preservation of mitochondrial function, resulting in apt cell response and adaptation. To our knowledge, no study has examined such hypothesis. Similarly, whether prevention of mitophagy leads to changes in the ischemic angiogenesis‐related gene expression and changes in the endothelial progenitor cell presence are unknown, especially in the females.

Ischemic angiogenesis and reestablishment of microcirculation within the IR‐damaged area represents a key vital process in the mobilization of immune and progenitor cells to repair/regenerate damaged tissue. Ischemic angiogenesis is especially critical for the coronary artery disease patients who are not the candidates for the standard revascularization techniques (Sim et al. 2002). Although there is much appreciation for the necessity of the ischemic angiogenesis, the role of mitochondrial homeostasis in the ischemic angiogenesis is still emerging (Ryan et al. 2015). Given the role of mitochondria in energy generation and redox balance (Ryan et al. 2015), the optimal mitochondrial function is not only necessary for the survival of individual cells but also can play a pivotal role in intercellular communication and adaptation to stress responses such as IR injury. Intercellular communication and prompt reestablishment of microcirculation assume utmost significance in the myocardial tissue where there is constant heavy workload is present. However, the role of mitochondrial dynamics, specifically the significance of excessive mitochondrial fission in the ischemic angiogenesis and microcirculation regulation after myocardial IR injury are unknown. Although the significance of mitochondrial fission inhibitors in amelioration of myocardial IR injury or infarction is well documented, the molecular angiogenic signatures of mitochondrial fission inhibitors during myocardial IR injury are completely unknown. Here, we hypothesized that suppression of mitochondrial fission during IR injury modulates angiogenic responses, thereby playing an important role in the regulation of post‐ischemic microcirculation. We found that there were a few specific changes in the angiogenic program in response to Mdivi‐1 treatment. The findings not only unraveled the role of mitochondrial dynamics in the ischemic angiogenesis and intercellular communication after IRI, but also useful in shaping the rational design of therapies aimed to prevent IR‐induced damage.

## Methods

### Animal models

Female mice with C57BL/6J background were procured from the Jackson labs at the age of 3 months and were given ad libitum access to standard chow and water and kept on a 12‐h light/12‐h dark cycle. A minimum number of five mice were used in each group [control/DMSO (Dimethyl sulfoxide) group and treatment group/Mdivi‐1 group] for the experiments except for the Figure 4. All the females were nulliparous. All the protocols were approved by the IACUC and are in line with the institutional guidelines.

### HL‐1 cell culture and CoCl2 treatment

HL‐1 cells were obtained from Millipore (Billerica, MA) and were cultured as reported before (Claycomb et al. 1998). To create hypoxia, the cells were treated with CoCl2 (Sigma, CAS# 7646‐79‐9.8.02540; St. Louis, MO) at the concentration of 300 μmol/L for 6 h. After 1 h, the cells were treated with DMSO or Mdvi‐1 (50 μmol/L). The mitochondria were visualized by incubating with Mito tracker RED CM‐H2XRos (Molecular probes) as per the instructions provided by the manufacturer. The live cell nuclei were stained with Hoechst 33342 (Molecular probes). The mitochondria in the fixed cells were visualized using confocal microscopy. The mitochondrial fragmentation index was calculated as reported previously (Senyilmaz et al. 2015).

### Creation of IR injury

Mice were subjected to 30 min of myocardial ischemia and 72 h of reperfusion, as described previously (Oyama et al. 2004) with the following modifications. Briefly, mice were anesthetized with pentobarbital sodium (70 mg/kg) and were intubated and ventilated with 100% oxygen. Ischemia was achieved by ligating the left anterior descending coronary artery (LAD), using a 6‐0 silk suture with a piece of PE‐10 tubing placed in the suture knot over the LAD. After visible confirmation of the pale colored ischemic zone, mice were left in position for 30 min. After 30 min of occlusion, reperfusion was initiated by releasing the ligature and removing the PE‐10 tubing. The disappearance of pale color below the site of ligation was checked to confirm the reperfusion. The chest wall was closed, and body temperature was maintained by use of a 37°C warm plate. The ventilator was disconnected after the animal recovered completely. Hearts were harvested 72 h later under TBE general anesthesia, flushed and washed in saline solution, separated ischemic and nonischemic portions, and were frozen immediately in liquid nitrogen (Veeranki et al. 2015). The separation was based on two criteria: right below the site of ligation and paler color of the tissue.

### Mdivi‐1 treatment

The drug mitochondrial division inhibitor, Mdivi‐1 (CAS # 338967‐87‐6), was purchased from Sigma, and was given as reported before (Givvimani et al. 2012). Briefly, the drug was administered through intraperitoneal route at a dose of 50 mg per kg body weight for a total of 5 days (2 days before the surgery, on the day of surgery, and 2 days after the surgery). The control group received an equivalent DMSO on the same days as with the treatment group.

### Angiogenic arrays

The relative expression profile of 53 mouse angiogenesis‐related proteins were semi‐quantitatively quantified, using a proteome profiler mouse angiogenesis antibody array (R&D Systems, ARY015; Minneapolis, MN). The array was hybridized with an equal amount of total protein (300 μg) isolated from the ischemic and nonischemic tissue lysates of DMSO and Mdivi‐1 treated mice hearts after IR injury. The assay was performed according to the manufacturer's protocol. We have performed two independent array sets for each group with five total animals per each group. The First array (four groups) was done with *n* = 3 samples by pooling equal protein amounts from the samples. In the next round, we have repeated the array with *n* = 2 samples (additional samples) by pooling equal protein amounts.

### Western blotting

The protein concentration was estimated, using the Bio‐Rad Bradford reagent. Equal amounts of total proteins (30 μg) were resolved, using SDS‐PAGE and transferred to polyvinylidene membranes. Membranes were probed with overnight primary antibody and 1 h of secondary antibody as described before (Veeranki et al. 2014). After probing the membranes with primary and secondary antibodies along with appropriate washes, chemiluminescence signal was detected using Bio‐Rad ChemiDoc™ XRS + System and Image Lab™ Software (Bio‐Rad, Hercules, CA). Antibodies: Connexin 43 is from Invitrogen (#355000; Carlsbad, CA); GAPDH is from Millipore (#374); Drp1 (#101270), Mfn2 (#100560), cluster of differentiation (CD31) (#1506), and Catalase (#50508) are from Santa Cruz biotech (Dallas, TX); superoxide dismutase 2 (SOD2) (#13141), Caspase3 (#9662) and pDrp1 (#6319) are from Cell Signaling Technologies (Danvers, MA); Tek (# 69753) is from Novus Biologicals and Bax (#182733; Littleton, CO) & Bcl2 (#692) are from Abcam (Cambridge, MA).

### Echocardiography

Left ventricular function was enumerated by transthoracic echocardiography, using a Vevo 2100 machine equipped with an MS550D transducer (Visual Sonics, Toronto, Canada) as described before (Veeranki et al. 2016). Briefly, the mice were anesthetized, using 3.5% isoflurane and depilated with Nair hair removal cream. Maintenance dose of isoflurane was 1.5% and heart rate was 450 ± 50 beats/min, while recording the cardiac parameters. Percentage ejection fraction (% EF) representing ventricular function, ventricular wall thickness, and chamber diameter were derived from the measurements during short‐axis M‐mode. LV fractional shortening (LVFS) was calculated according to the following equation: LVFS = ([LVEDD−LVESD]/LVEDD) × 100. Wherever possible, all of the physiological measurements were collected on the same period of the day to minimize variation.

### Q‐PCR

Total RNA was isolated, using TRIzol reagent (Thermo Fisher Scientific, Grand Island, NY). An equal amount of total RNA was subjected to cDNA synthesis with the usage of RT‐PCR reagents from Qiagen (Germantown, MD). The SYBR‐green dye was used to quantify the amplicons. The expression levels of different endothelial progenitor markers were assayed using the following primer pairs (given as 5′ to 3′ order): Tek‐F1: GAGTCAGCTTGCTCCTTTATGG,Tek‐R2:AGACACAAGAGGTAGGGAATTGA; KdrF1:TTTGG CAAATACAACCCTTCAGA, Kdr‐R1: GCAGAAGATACTGTCACCACC; Flt4‐F1:ACAGAAGC TAGGCCCTACTG, Flt4‐R1: ACCCACATCGAGTCCTTCCT.

### Statistical analysis

All values are exhibited as mean ± SE. Densitometry analysis was performed, using Image Lab software. *P* values of <0.05 were considered significant. Statistical analysis was done using Primer of Biostatistics 7.0 (McGraw‐Hill, New York, NY). Comparisons were made by one‐way ANOVA (analysis of variance) followed by Bonferroni correction for multiple groups.

## Results

### Mdivi‐1 treatment inhibited hypoxia‐induced mitochondrial fragmentation

**Figure 1:**
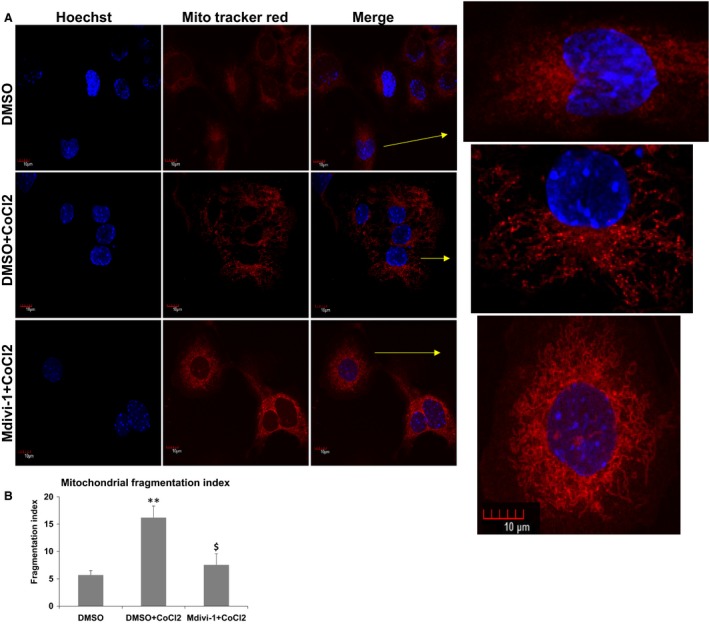
Mitochondrial division inhibitor 1 (Mdivi‐1) prevents CoCl2‐induced mitochondrial fission in HL‐1 cells. (A) Confocal images depicting mitochondrial structures (100x) in HL‐1 cells. The representative cells were enlarged from each group. (B) Quantification of mitochondrial fragmentation index from three different treatments with *n* = 15 cells. * is *P* < 0.05 versus DMSO and $ is *P* < 0.05 versus DMSO + CoCl2.

To test if the Mdivi‐1 can protect hypoxia‐induced mitochondrial fission in our hands as demonstrated thoroughly before (Ong et al. 2010; Bhatt et al. 2013; Hong et al. 2013; Kim et al. 2014; Han et al. 2015; Twaroski et al. 2015; Pagliuso et al. 2016; Tanner et al. 2017), we have treated the HL‐1 cells with the CoCl2 and then with the Mdivi‐1. As depicted in the Figure 1, the Mdivi‐1 treatment protected CoCl2‐induced mitochondrial fission very effectively. Further, there was visible enhancement in the filamentous mitochondrial structures as early as 5 h. These observations further confirmed the previous findings and corroborated the effectiveness of the drug.

### Mdivi‐1 treatment ameliorated cardiac dysfunction associated with IR injury in the female mice

**Figure 2:**
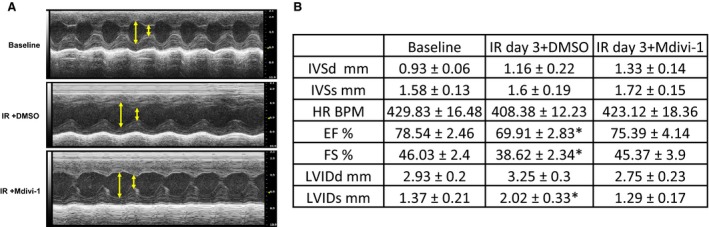
Evaluation of left ventricular function after ischemia reperfusion injury (IR injury) and mitochondrial division inhibitor 1 treatment. (A) Representative M‐mode echocardiography images from each are presented. Arrows indicate diastolic (longer) and systolic (shorter) chamber lengths. (B) Various echocardiographic parameters for each group are presented as mean ± SE. * is *P* < 0.05 versus baseline; *n* = 5.

Left ventricular function was evaluated before IR injury (baseline) and on the day 3 of IR injury in both control (DMSO) and treatment groups (Mdivi‐1) by collecting M‐mode echocardiograms. As shown in Figure 2, after IR injury, control group exhibited significant attenuation in the fractional shortening and ejection fractions in parallel with enlargement of LV inner diameter (Fig. 2B). Contrary to the control group mice, Mdivi‐1 treated mice did not exhibit significant changes in the heart function after IR injury. These findings implied that Mdivi‐1 treatment is associated with containment of IR‐induced injury.

### Mdivi‐1 treatment‐mediated changes in the angiogenic profile

**Figure 3:**
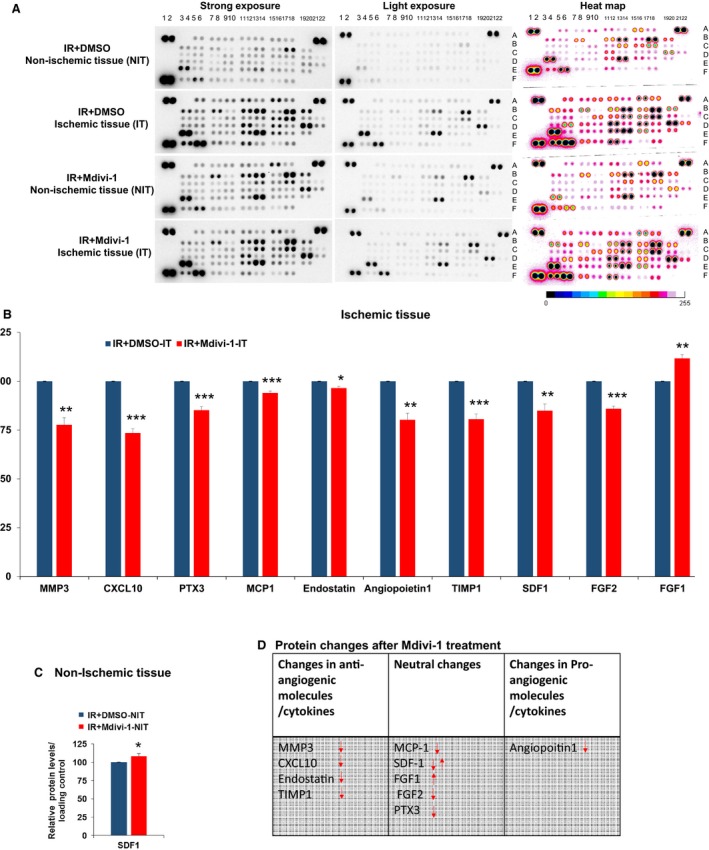
Angiogenic arrays showing the differences in angiogenic profiles after mitochondrial division inhibitor 1 treatment and ischemia reperfusion injury (IR injury). (A) Representative images of arrays indicating the differences in each group. Circles compare very obvious changes. (B) and (C) bar graphs presenting quantification of relative differences in the levels of various angiogenesis‐related proteins. (D) Table is showing the classification of molecular changes and their direction (arrows up increase and arrows down decrease). * is *P* < 0.05 versus control treated tissue. **P < 0.01 and ***P < 0.001; *n* = 5. IT for Ischemic tissue. NIT for Non‐ischemic tissue.

Next, to decipher the changes in the angiogenic profile, we have assayed the relative levels of 53 different proteins that have been implicated in the angiogenic process. The tissue lysates were from the ischemic and nonischemic tissues separated from the same heart after the IR injury. As observed in the Figure 3, several protein changes could discriminate ischemic tissues from the nonischemic tissues. The prominent changes among these are coagulation factor (CFIII) (A15,16), CXCL10 (D5,6), HGF (C11,12), IGFBP (Insulin‐like growth factor‐binding protein)‐1,2,3&10 (C13,14; C15,16; C17,18 & B3,4), MCP‐1 (D11,12), matrix metalloproteinase (MMP3) (D15,16) (Kelly et al. 2008), MMP9 (D19,20) (Romanic et al. 2002), osteopontin (E3,4), plasminogen activator inhibitor‐1 (PAI1) (F5,6), Pentraxin3 (E11,12), PIGF2 (E15,16), stromal cell‐derived factor 1 (SDF1) (F3,4), and tissue inhibitor of metalloproteases (TIMP1) (F11,12). All of these were previously reported to be elevated after myocardial infarction or IR injury (please refer to the discussion below). Hence, these profiles will serve as a reference for the angiogenic program in the females after the IR injury.

In addition, we observed specific and reproducible changes in angiogenic profiles after the Mdivi‐1 treatment. As depicted in Figure 3B, these changes represent a significant deviation from the control treated ischemic tissue. These molecules include MMP3, CXCL10, Pentraxin 3 (PTX3), monocyte chemoattractant protein‐1 (MCP1), endostatin, angiopoietin‐1, TIMP1, SDF1, and fibroblast growth factor (FGF1 and 2). Based on the literature, we classified these changes as a pro, neutral, and anti‐angiogenic molecules (Fig. 3D). Based on this classification, it can be inferred that overall, Mdivi‐1 treatment led to a positive angiogenic response with minor changes in the couple of the pro‐angiogenic molecules. Certain molecular changes may not alter the overall angiogenesis phenotype (dubbed as neutral changes). For instance, the reduction in the FGF2 levels was compensated by the FGF1 level enhancement. Likewise, though the SDF1 levels were reduced in the ischemic tissues after the Mdivi‐1 treatment, the levels were enhanced by the treatment in the nonischemic portions of the heart (Fig. 3C).

### Mdivi‐1 treatment enhanced endothelial progenitor cell (EPC) markers in the ischemic tissues after the IR injury

**Figure 4:**
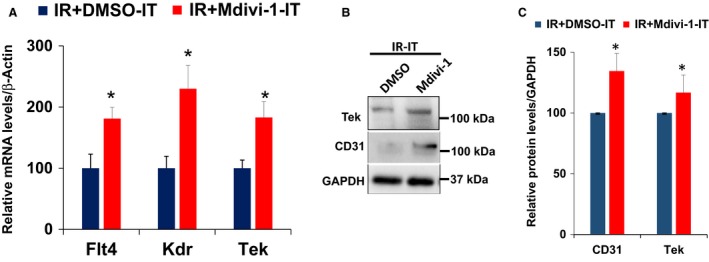
Assay of markers of endothelial progenitor cells. (A) Bar graph presenting the Q‐PCR based quantification of mRNA levels. (B) Western blot images are presenting the differences in the CD31 and Tek levels. (C) Bar graph with quantification of the Western blot data. * is *P* < 0.05 versus control treated ischemic tissue; *n* = 4.

To know, if the above changes in the angiogenic profiles could contribute to the angiogenic process, we assayed for the CD31 levels, a known marker for endothelial cells. As presented in the Figure 4, Mdivi‐1 treatment significantly enhanced the CD31 levels compared to the control treated animals after the IR injury. We further examined the other markers of endothelial progenitor cells in the ischemic tissues by Q‐PCR. As presented in the Figure 4, Mdivi‐1 treatment significantly upregulated the levels of the endothelial‐specific receptor tyrosine kinase (Tek), fMS‐like tyrosine kinase 4 (Flt4) and kinase insert domain protein receptor (Kdr) in the ischemic tissues. We confirmed the higher Tek marker presence by the western blotting as well. These results further suggested that the overall betterment in the angiogenic profile changes after the Mdivi‐1 treatment led to the enhanced angiogenic potential.

### Mdivi‐1 treatment prevented Drp‐1 function

**Figure 5:**
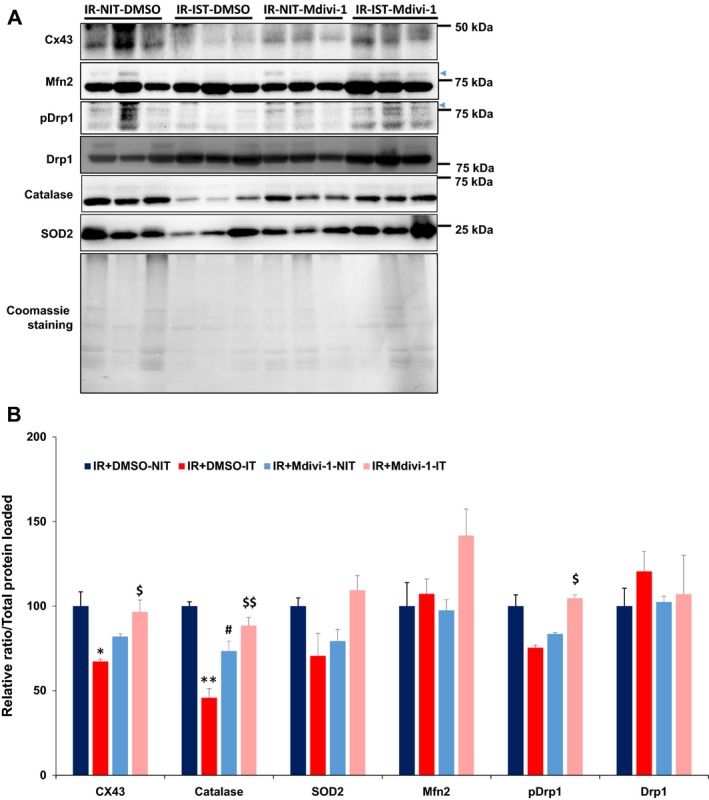
Quantification of changes in different protein levels by western blotting. (A) Images are showing the relative levels of the various proteins among the groups. (B) bar graphs are presenting quantification of relative differences in the levels of various proteins. **P < 0.01 versus DMSO‐NIT, $$ *P* < 0.01 versus DMSO‐IT, ## *P* < 0.01 versus DMSO‐NIT. Single symbol represents *P* < 0.05; *n* = 3. NIT for nonischemic tissue; IT for Ischemic tissue.

To test the regulation of mitochondrial biogenesis after the IR injury and after Mdivi‐1 treatment, we have assayed for the levels of Drp1, Drp1 phosphorylation status (S637), and Mfn2 levels. As showed in the Figure 5 (A and B), the levels of Drp1 were modestly upregulated after IR injury when compared to that of the nonischemic tissues from the same heart. Interestingly, IR injury, in conjunction with the enhanced Drp1 levels, also reduced its inhibitory phosphorylation at S637 significantly (Fig. 5). In contrast to the controls, ischemic tissues from the Mdivi‐1 treated group exhibited significantly higher levels of Drp1 S637 phosphorylation (Fig. 5B). The Mfn2 levels were also found to be elevated after Mdivi‐1 treatment in the IR injured tissue, indicating very effective prevention of mitochondrial fission.

### Mdivi‐1 treatment rescued connexin 43 (Cx43) downregulation after IR injury

As preservation of intercellular coupling through Cx43 play an integral role in the maintenance of ultrastructure and attenuation of IR injury (Zhao et al. 2013), we assayed for the Cx43 levels. As shown in Figure 5A and B, the Cx43 levels were significantly downregulated in the ischemic zone of the DMSO treated group, but the Mdivi‐1 treatment significantly alleviated such downregulation/degradation. Interestingly, Mdivi‐1 treatment was inclined to lower the Cx43 levels in the nonischemic tissues, but the differences were not statistically significant (*P* = 0.08).

### Mdivi‐1 treatment enhanced antioxidant capacity after IR injury

As IR injury was demonstrated to elevate oxidative damage, next, we tested if there were any significant changes in the two important antioxidant enzyme levels: SOD2 and catalase. As presented in the Figure 5A and B, the catalase levels were significantly lower in the ischemic tissues of DMSO treated group. Importantly, the treatment with Mdivi‐1 significantly ameliorated such decline in the catalase levels in the ischemic tissues. Interestingly, the SOD2 levels followed the similar trend as observed with the catalase, but the differences were not significantly different. Mdivi‐1 treatment also significantly attenuated the catalase levels in nonischemic tissues. These data together imply that Mdivi‐1 treatment is associated with preservation of antioxidant enzymes, especially in the ischemic tissues.

### Mdivi‐1 treatment suppressed apoptosis activation

**Figure 6:**
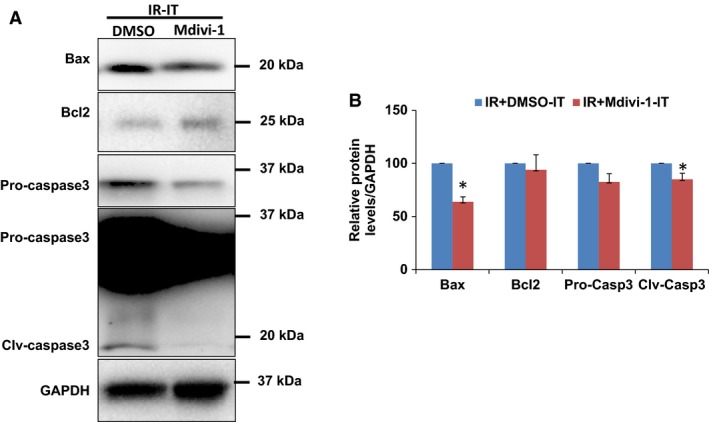
Quantification of various apoptosis activating proteins by Western blotting. (A) representative western images are presented. (B) quantification of relative protein levels. * is *P* < 0.05 versus control treated ischemic tissue; *n* = 4.

Next, to examine the potential mechanisms of better angiogenic response and higher EPCs presence after the treatment, we have assayed for the mitochondrial‐mediated apoptosis regulators and effector caspase‐3 activation. As presented in Figure 6, the Bax/Bcl2 ratio elevation was ameliorated with the Mdivi‐1 treatment. Further, the treatment was effective in suppression of the caspase‐3 activation. These findings together implied that the Mdivi‐1 treatment is associated with the lower apoptotic profile, thereby enabling the mounting of better angiogenic response, better cell survival and functional improvements.

## Discussion

Proper function and intactness of mitochondria are essential for energy production, Ca^2+^ homeostasis, redox status regulation, and apoptosis suppression. It was noted that IR injury causes cell death and enhancement of ischemic lesion due to the raise in oxidative radicals, poor Ca^2+^ homeostasis, enhanced MTP opening and mitochondrial failure (Webster 2012). Consequently, drugs that inhibit abnormal MTP opening and abnormal mitochondrial fission were shown to reduce IR injury (Ong and Hausenloy 2010; Ong et al. 2010; Disatnik et al. 2013). Recently it has been demonstrated that alterations in the mitochondrial function resulted in differential transcriptome profiles (Picard et al. 2015). Despite such progress, the effects of inhibition of mitochondrial fission on the angiogenic responses after myocardial IR injury are known especially in the females. Angiogenesis/neovascularization following IR injury is crucial in the regulation of infarct size and cardiac remodeling, which are the key determinants of clinical outcome after the heart attacks. Angiogenesis and its regulators during the ischemic heart disease are particularly important in the patients with coronary heart disease, who do not respond satisfactorily to the current recommended drugs, and revascularization techniques (Kastrup 2010). In the current study, we used the known inhibitor of mitochondrial fission regulator (Drp1), Mdivi‐1, to modulate mitochondrial dynamics and assayed for the changes in the angiogenic responses after IR injury (30 min of ischemia and 72 h of reperfusion) in the female mice. We have used Mdivi‐1 treatment starting two days prior to the IRI, because the ROS burst has been noted to peak within 5–6 min after reperfusion and cell death has been observed as early as 30 min/even less after the IRI (Becker 2004).

The Mdivi‐1 treatment attenuated IR‐induced deficiencies in heart function. Particularly, improvements in fractional shortening and ejection fractions were observed after IR injury. These results suggested improved cardiac contractile function and force generation, which are the indirect indicators for improved mitochondrial function. Furthermore, Mdivi‐1 treatment also ameliorated abnormal left ventricle dilatation after IR injury (Fig. 2). These findings implied that Mdivi‐1 treatment is associated with the successful reduction in IR‐induced damage.

Our unbiased, simultaneous, multiple (53) angiogenesis‐related protein profiling revealed significant changes in the levels of both pro and anti‐angiogenic regulators after IR injury. The profiling also revealed significant elevation in the previously established markers of ischemic injury in both the treatment groups (DMSO and Mdivi‐1) in the ischemic tissues when compared to the levels of these molecules in the nonischemic portions of the heart. This group (Fig. 3A) included CFIII (A15,16) (Erlich et al. 2000), CXCL10 (D5,6) (Frangogiannis 2004), HGF (C11,12) (Nakamura et al. 2000), IGFBP‐1,2,3&10 (C13,14; C15,16; C17,18 & B3,4) (Chi and Karliner 2004), MCP‐1 (D11,12) (Gonzalez‐Quesada and Frangogiannis 2009), MMP3 (D15,16), MMP9 (D19,20) (Romanic et al. 2002), Osteopontin (E3,4) (Singh et al. 2010), PAI1 (F5,6) (Zaman et al. 2007), Pentraxin3 (E11,12) (Salio et al. 2008), PIGF2 (E15,16) (Iwama et al. 2006), SDF1 (F3,4) (Liehn et al. 2011) and TIMP1 (F11,12) (Cavusoglu et al. 2006). The following can be inferred from these findings: (1) Successful creation of IR injury, (2) Efficient separation of ischemic and nonischemic tissue portions from the same heart and (3) Mdivi‐1 treatment does not alter the majority of the angiogenesis regulators, but might involve selective modulations.

There were selective changes in the levels of angiogenesis modulators after the treatment (Fig. 3). Reduction in the MMP3 levels has many implications in the angiogenesis and in limiting the damage after IR injury. MMP3 potently cleaves all forms of VEGF‐A (heparin bound and unbound) and depletes the VEGF reservoir in the tissues, thus adversely affects the angiogenic process. Further, the cleaved VEGF, though retains biological angiogenic activity, its effects on the capillary formation are distinct from the uncleaved VEGF. The former stimulated dilated unbranched abnormal capillaries, whereas the later stimulated normal and branched capillary formation (Lee et al. 2005). In addition, the MMP‐3 level reduction might well correlate with the decline in the activated MMP9 levels, as MMP‐3 is involved in cleavage of pro‐MMP9 (Defamie et al. 2014). It has been proposed that MMP9 targeting confers IR protection (Romanic et al. 2002). Recently, it has been suggested that MMPs might degrade CX43 levels and might alter cell to cell communication (Givvimani et al. 2014; De Bock et al. 2015). Further, excess MMP‐3 might also cleave intracellular targets as well (Azevedo et al. 2014). As there was a negative correlation with regard to the MMP3 levels and Cx43 levels in the ischemic tissues with and without Mdivi‐1 treatment, it is most likely that MMP3 level reduction might have a protective effect on the IR‐induced Cx43 degradation. Interestingly, Mdivi‐1 treatment also significantly suppresses TIMP1 levels in the ischemic tissues, hence, may provide beneficial myocardial remodeling after IR injury. As there was also a reduction in the MMP3 levels, the main activator of other MMPs, the decline in the TIMP1 may be to fine tune it levels to the MMP3 level reduction/other MMPs activation in a well‐coordinated response (Fig. 3B). Levels of TIMP1 were positively correlated with MI mortality rates (Cavusoglu et al. 2006) and were negatively correlated with angiogenesis (Ikenaka et al. 2003; Reed et al. 2003). In addition to TIMP1, MMP3 was also reported to predict the risk of MI (Cavusoglu et al. 2016). However, it was also emphasized that lack of TIMP1 also exacerbates LV remodeling after MI (Creemers et al. 2003). Hence, optimal regulation of TIMP1 is crucial for limiting IR‐induced damage. In this context, Mdivi‐1 treatment may prove to be beneficial as it reduces the TIMP1 levels in the ischemic tissues.

Apart from MMP3 and TIMP1, Mdivi‐1 also suppressed other potent anti‐angiogenic molecules (Fig. 3). CXCL10 produces angiostatic and inflammatory effects in the mice (van den Borne et al. 2014). Likewise, Endostatin is a well‐known anti‐angiogenic factor (Dobryansky et al. 2004; Panchal et al. 2004), which was suppressed by the Mdivi‐1 treatment. Moreover, we were able to observe enhanced endothelial progenitor cell markers (CD31, Flt4, Kdr and Tek) (Fadini et al. 2012; Lui et al. 2013; Kawakami et al. 2015) enhancement suggestive of endothelial progenitor cell recruitment, an essential part in the neo‐angiogenesis. Previous studies have identified these markers are highly specific to the endothelial progenitor cells (Fadini et al. 2012; Lui et al. 2013; Kawakami et al. 2015). All these results suggest Mdivi‐1 treatment is beneficial in post‐IRI angiogenesis. In addition to the proangiogenic responses noted above, Mdivi‐1 treatment also led to changes in certain cytokines, which are neutral in nature (Fig. 3D). For instance, FGF2 downregulation is associated with FGF1 upregulation. Both FGF1 and FGF2 are pro‐angiogenic in nature (Fernandez et al. 2000; Engel et al. 2006; Virag et al. 2007; Kastrup 2010). Interestingly, the suppression in the apoptotic activation (Fig. 6) could also promote myofibroblast proliferation. However, prevention of adverse matrix remodeling through suppression of MMP3 and preservation of intercellular communication and cardiomyocyte/endothelial structural features through better retainment of Cx43 levels could potentially hinder such fibroblast proliferation. The Mdivi‐1 treatment also altered the molecular pattern of the SDF‐1 molecule. Levels of SDF‐1 were elevated in the nonischemic portions, whereas its levels were suppressed in the ischemic portions. Currently, the significance of such variations is unknown, but SDF‐1/CXCR4 axis has a dual role in the myocardial recovery (Liehn et al. 2011; Scirica et al. 2013; White et al. 2013; Weir et al. 2014; Chung et al. 2015; Zhong and Rajagopalan 2015). Mdivi‐1 treatment also resulted in a decline in the MCP‐1 levels. According to a previous study, MCP‐1 deficiency might reduce myofibroblast proliferation, but is not associated with changes in the angiogenesis or infarct size (Gonzalez‐Quesada and Frangogiannis 2009). Similarly, PTX3, whose deficiency resulted in enhanced MI damage with decreased number of capillaries (Salio et al. 2008), was also suppressed by the Mdivi‐1 treatment. However, PTX3 was also shown to inhibit angiogenesis through FGF antagonism (Inforzato et al. 2010). In contrast to the above changes, Mdivi‐1 treatment suppressed a known pro‐angiogenic molecule, Angiopoietin1. It was reported that Angiopoietin1 enhancement during the acute phase of MI enhances capillary density and reduces infarct size (Takahashi et al. 2003). Currently, the significance of this finding is unclear. Other positive changes in the pro and anti‐angiogenic factor presence might have reduced the requirement for the Angiopoietin1.

A recent report has suggested that Mdivi‐1 treatment leads to inhibition of mitochondrial respiration as well (Qian et al. 2014). Our data (Fig. 3C) about the SDF‐1 levels in the nonischemic tissues also indicates some state of hypoxia, as SDF‐1 induction is mainly associated with hypoxia (Krock et al. 2011). Whether such inhibition of respiration/hypoxia state is also involved in Mdivi‐1‐mediated cardioprotection, need to be addressed further.

We also examined the status of the mitochondrial biogenesis regulators Mfn2 and Drp1 in different groups. As presented in Figure 5, there were significant changes in the levels of Mfn2 between groups, implying that the mitochondrial function was preserved after ischemia with the treatment. Interestingly, we found significant elevation in the Drp1 levels in the ischemic zones (Fig. 5). Given such upregulation, usage of mitochondrial fission inhibitors would be mandatory to curtail the IR damage. It has to be noted that the Mdivi‐1 treatment also tends to enhance (not significantly) the Drp1 levels. One potential explanation for such a phenomenon is that compensatory mechanisms were in place to boost the Drp1 levels during the inhibition. We further verified the levels of Drp1 phosphorylation status (S637), which inhibits its migration to the mitochondria, thereby leading to inhibition of fission. As showed in the Figure 5, the Drp1 phosphorylation (pDrp1) was significantly suppressed in the ischemic zone of the control treated mice. Contrary to this, pDrp1 levels were elevated after Mdivi‐1 treatment. However, such elevation was dwarfed by the presence of higher levels of total Drp1 in the ischemic zone (Fig. 5). Nonetheless, the results imply that there was lower cytosolic Ca^2+^ levels (leads to reduced calcineurin activity, which dephosphorylates Drp1 (Cereghetti et al. 2008)) or higher PKA or Cdk1 activities (inhibits Drp1 through S637 phosphorylation) or both after the Mdivi‐1 treatment. Overall, Mdivi‐1 treatment promoted suppression of fission and enhancement of fusion as well.

In parallel with the better angiogenic profile, Mdivi‐1 treatment also prevented the degradation of the Cx43 in the ischemic zones. Connexins, especially Cx43, are implicated efficient tube formation (Dhein et al. 2015) during angiogenesis. As there was increased presence of endothelial progenitor cells after Mdivi‐1 treatment (Fig. 4), the neoangiogenesis might have contributed to Cx43 elevation. Further support for such a possibility comes from the studies that measured post‐ischemic levels of Cx43 (degradation, Fig. 4; He et al. 2015) following IR injury. Alternatively, the preservation of Cx43 could be due to the prevention of cell death and preservation of architectural integrity. Furthermore, prevention of mitochondrial fission could have also ameliorated the Cx43 degradation, as Cx43 is also localized in the mitochondria and plays a key role in intermitochondrial communication. Support for such a possibility comes from the study that showed Cx43 levels and mitochondrial morphology is interlinked (Trudeau et al. 2012). Other studies also proposed that ischemic preconditioning mediates cardiac protection involve Cx43 upregulation (Boengler et al. 2005). In spite of Cx43's role in coupling of cardiomyocytes and possibly the mitochondria for functional coordination and morphological integration, it was proposed that Cx43 presence may cause larger infarct size and possibly associated with the deterioration in the cardiac function, following the ischemic injury (Kanno et al. 2003). It is tempting to speculate that reduction in MMP3 levels might have contributed to CX43 preservation especially in the mitochondria (as discussed above). Taken together, Mdivi‐1‐mediated Cx43 preservation/elevation could be the reason in the preservation of the heart function following IR injury.

Our assay for the antioxidant enzyme levels revealed considerable downregulation in the SOD2 levels after IR injury, which was ameliorated with the mitochondrial fission inhibition. Previous studies also reported that SOD activity is diminished after MI(Ikonomidis et al. 2005). As SOD2 is localized mainly in the mitochondria, this finding further supports the notion that fission inhibition maintained the mitochondria in a healthier condition after IR injury. Interestingly, we noted dramatic changes in the catalase levels after IR injury and after Mdivi‐1 treatment. It was demonstrated that catalase levels enhancement is associated with better recovery following the MI (Pendergrass et al. 2011). Collectively, inhibition of mitochondrial fission effectively suppressed the IR‐induced decline in the catalase levels and SOD2 levels, which further aids in the recovery process and mitigation of IR‐induced acute oxidative damage.

**Figure 7:**
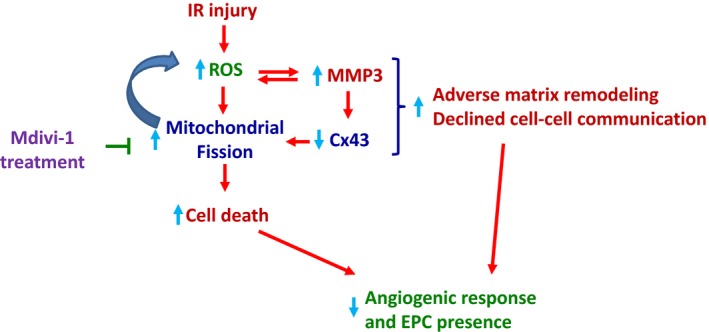
Schematic diagram summarizing the overall findings.

In conclusion, Mdivi‐1 treatment conferred amelioration of the IR injury‐induced functional deterioration through the following (Fig. 7): (1) Through apoptosis inhibition, which resulted in the better mounting of angiogenic profile and EPC survival. (2) Through preservation/elevation of Cx43‐mediated intercellular or interorganellar communication possibly through MMP3 level suppression. (3) Through maximizing the antioxidant capacity. Interestingly, all these modulations (levels of ROS, mitochondrial fission and MMP3) are interlinked, and inhibition of mitochondrial fission may result in a positive directional response, i.e., improved function after IR injury. For example, enhanced reactive oxygen species (ROS) were demonstrated to cause mitochondrial fission and MMP3 induction (Del Carlo et al. 2007; Wu et al. 2011). The enhanced mitochondrial fission could further enhance ROS production through reduced mitochondrial function (Yu et al. 2008). Likewise, MMP3 could further enhance ROS production (Radisky et al. 2005). These feedback upregulation in the ROS production enhancement overwhelms antioxidant capacity leading to membrane permeability pore opening and initiation of mitochondrial‐mediated apoptotic pathway (Webster 2012). The Mdivi‐1 treatment breaks this vicious cycle by suppressing fission, ROS and MMP3 levels, thereby inhibits apoptosis, adverse matrix remodeling and decline in the intercellular communication. All these beneficial influences culminate into the mounting of better angiogenic response, EPC presence and angiogenic potential. This is the first study to reveal the potential benefits of the acute inhibition of mitochondrial fission in the angiogenic profile after the IR injury. The findings will aid in the rapid translation of mitochondrial fission inhibition strategies i.e., Mdivi‐1 use into the clinic.

### Limitations

We have focused only on the acute changes (day 3 after the IR injury).

## Conflict of Interest

The authors declare that there is no duality of interest associated with this manuscript.
